# Characterization and phylogenetic analysis of the chloroplast genome of *Solanum pseudocapsicum* (Solanaceae)

**DOI:** 10.1080/23802359.2024.2410442

**Published:** 2024-09-30

**Authors:** Yongle Liu, Xuan Tang, Aihua Deng, Huan Li, Yulong Xiao, Wenyan Zhao, Lixuan Xiang, Yi Liu, Zui Yao, Xingyu Zeng, Zhitian Du, Rongjie Huang, Hanbin Yin, Kerui Huang

**Affiliations:** Hunan Provincial Key Laboratory for Molecular Immunity Technology of Aquatic Animal Diseases, College of Life and Environmental Sciences, Hunan University of Arts and Science, Changde, Hunan, China

**Keywords:** *Solanum pseudocapsicum*, chloroplast genome, phylogenetic analysis

## Abstract

*Solanum pseudocapsicum* Linnaeus 1753, a popular indoor potted plant known for its ornamental fruits, had its chloroplast genome sequenced in this study to determine its phylogenetic relationship with other related species and to construct a phylogenetic analysis tree. The research findings are as follows: 1. The chloroplast genome of *S. pseudocapsicum* comprises a large single-copy (LSC) region of 86,260 base pairs, a small single-copy (SSC) region of 18,325 base pairs, and two inverted repeat (IR) regions, each measuring 25,390 base pairs in length. 2. The G + C content of the entire chloroplast genome is 37.59%, with the highest G + C content found in the IR regions, reaching 43.03%; followed by the LSC region, which has a G + C content of 35.68%; and the lowest in the SSC region, with a G + C content of 31.53%. 3. The genome contains 127 genes, including 82 protein-coding genes, 37 tRNA genes, and 8 rRNA genes, with 18 genes duplicated in the IR regions. 4. Phylogenetic analysis revealed that *S. pseudocapsicum*, *Solanum betaceum*, *Solanum laciniatum*, and *Solanum nitidum* are genetically closely related and are located on the same branch of the phylogenetic tree, indicating a close relationship among them. This study provides a foundation for the identification, classification, and exploration of genetic diversity within the *Solanum* genus.

## Introduction

**Figure 1.**
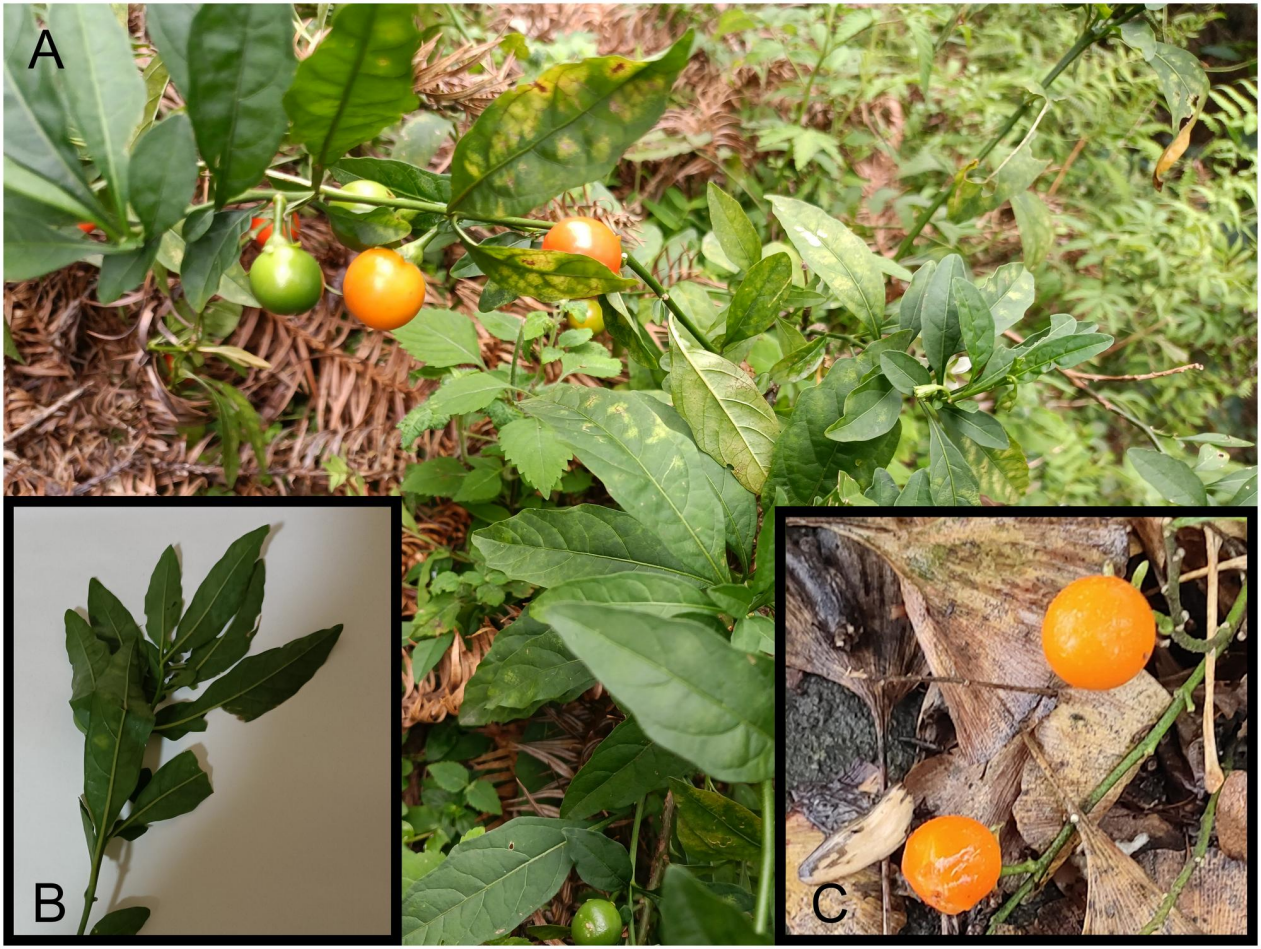
Picture of the collected sample of *Solanum pseudocapsicum*. Note: A is an overall photo of *Solanum pseudocapsicum* and its habitat; B is a photo of the leaves; C is a photo of the fruit. The picture is self-taken by Hanbin Yin; the sample was collected from the southern slope of Mount Hengshan in Nan Yue District, Hengyang City, Hunan Province (27°26′46.06ʺN, 112°71′90.94ʺE, 362m). The leaves of *S. pseudocapsicum* range from narrowly oblong to lanceolate, with a cuneate base, and the fruiting pedicel is expanded at the apex. The berries are initially green and turn orange-red upon maturity. Yongle Liu, Xuan Tang, and Huan Li identified this species with the assistance of Du Deng, an expert in plant taxonomy at Hunan University of Arts and Science.

*Solanum pseudocapsicum* Linnaeus 1753 (Figure 1) is a small shrub belonging to the Solanaceae family, genus *Solanum*, and the erect branch, native to South America and cultivated in Anhui, Jiangxi, Guangdong, Guangxi, and other regions of China. *S. pseudocapsicum* possesses high ornamental value and is a rare choice for ornamental fruiting plants during the off-season of New Year’s Day and Spring Festival, with its fruiting period lasting up to three months or more from fruit setting to maturity, making it one of the longest fruiting varieties among potted ornamental fruiting plants (Wu et al. 1994). Additionally, *S. pseudocapsicum* has significant medicinal value, as it can be used to treat boils, gonorrhea, and acute abdominal pain (Xu et al. 2018), and exhibits antibacterial, antiviral, antispasmodic, and antihypertensive properties (Vijayan et al. 2004). Moreover, the roots of *S. pseudocapsicum* can be used medicinally for promoting blood circulation and relieving pain, primarily for the treatment of lumbar muscle strain and sprains. Furthermore, studies have shown that the total alkaloid fraction of unripe *S. pseudocapsicum* fruits has significant *in vitro* cytotoxicity and antitumor properties (Aliero et al. 2006), and the ethyl acetate extract from *S. pseudocapsicum* seeds has also demonstrated good anti-feeding and insecticidal activities (Jeyasankar et al. 2012). Despite the immense ornamental and pharmaceutical value of *S. pseudocapsicum*, its phylogenetic development has not been thoroughly researched, and its chloroplast genome has not been fully elucidated, with limited molecular-level information currently available. Therefore, it is necessary to conduct phylogenetic studies and explore the chloroplast genome of *S. pseudocapsicum*. This study characterizes (sequences, assembles, and annotates) the chloroplast of *S. pseudocapsicum* and conducts a phylogenetic analysis, which is conducive to further exploration of the phylogenetic development of *S. pseudocapsicum* and provides an important theoretical reference for uncovering its potential value.

## Materials

The *S. pseudocapsicum* leaf samples were collected from the southern slope of Mount Hengshan in Nan Yue District, Hengyang City, Hunan Province (27°26′46.06ʺN, 112°71′90.94ʺE, 362 m). These specimens are currently well preserved at Hunan University of Arts and Science, and can be consulted in its herbarium (contact: Kerui Huang, huangkerui@huas.edu.cn, voucher number SHY002). Yongle Liu, Xuan Tang, and Huan Li identified this species with the assistance of Du Deng, an expert in plant taxonomy of Hunan University of Arts and Science.

## Methods

The procedure for extracting and sequencing the complete genomic DNA was carried out as described by Zhang et al. (2023). Total DNA was extracted from liquid nitrogen-preserved fruit samples using the DNAeasy Plant Tissue Extraction Kit (Beijing Tiangen Biotech Co. Ltd.). Sequencing was performed on the Illumina HiSeq 2500 platform (Personal Biotechnology Co. Ltd.) after library construction. Following the removal of low-quality reads with fastp (Chen et al. 2018), a total of 606,915,981 reads was discarded. Subsequently, GetOrganelle v1.7.5 (Jin et al. 2020) was employed to perform a de novo assembly of the *S. pseudocapsicum* chloroplast genome. To complete the genomic study, CPGAVAS2 (Shi et al. 2019) was utilized for annotating the chloroplast genome, and CPGView (1kmpg.cn/cpgview/) was used to visualize the genome map. For the phylogenetic analysis, we procured 69 chloroplast genomes that are closely related to *S. pseudocapsicum* from GenBank. We extract 68 common protein-coding genes across all genomes and aligned each gene separately with MAFFT v7.313 (Rozewicki et al. 2019). The genes were then processed with Gblocks 0.9b and concatenated into a super-gene for each species, incorporating all genes (Guo et al. 2022). The maximum-likelihood phylogenies were reconstructed using IQ-TREE v1.6.8 (Nguyen et al. 2015) with the TVM+F + I + G4 model, supported by 5000 ultrafast bootstrap replicates.

## Results

**Figure 2.**
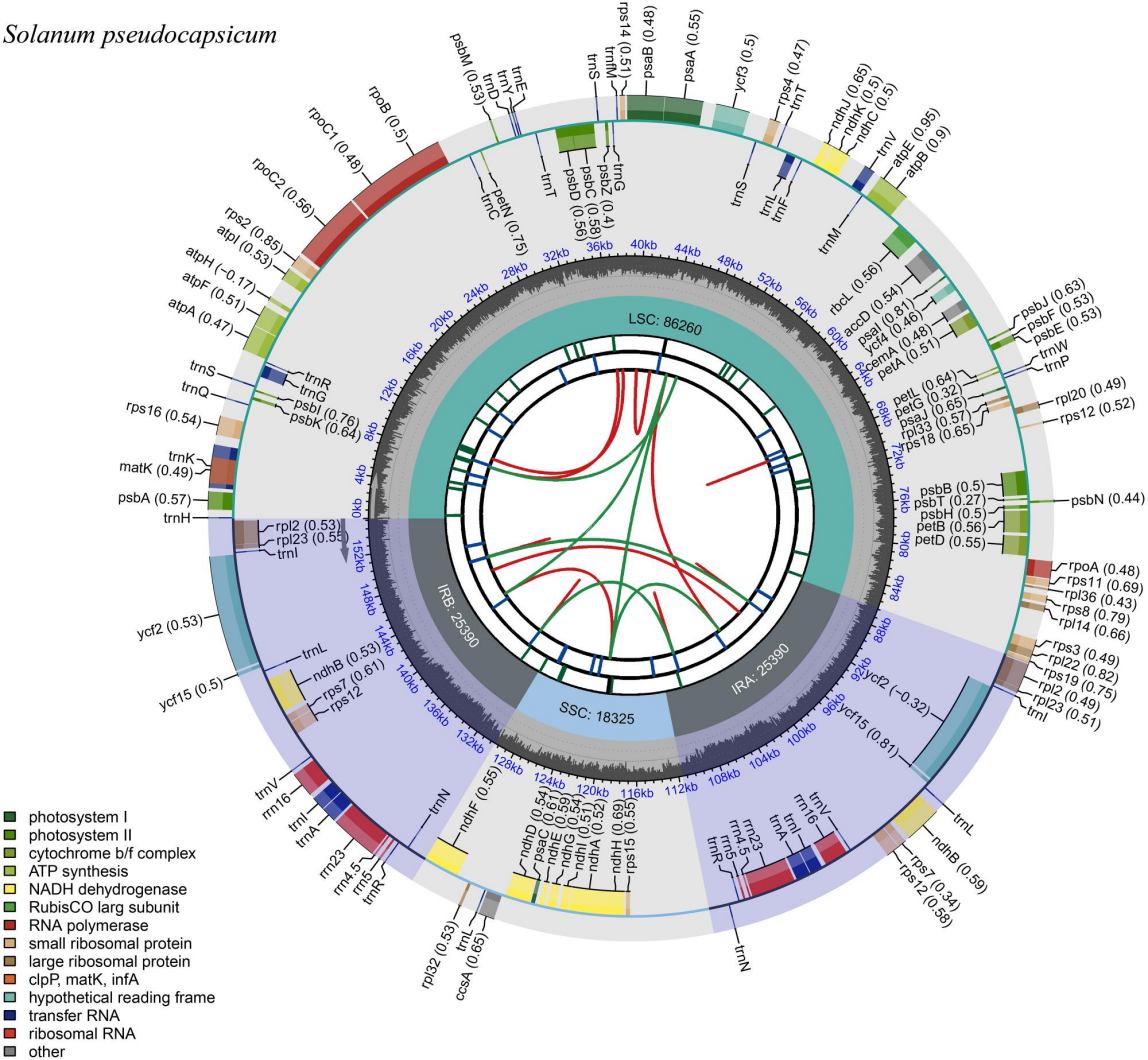
Gene map of the *Solanum pseudocapsicum*. From the center outward, the first track indicates the dispersed repeats; the second track shows the long tandem repeats as short blue bars; the third track shows the short tandem repeats or microsatellite sequences as short bars with different colors; the fourth track shows small single-copy (SSC), inverted repeat (IRA and IRB), and large single-copy (LSC) regions. The GC content along the genome is plotted on the fifth track; the genes are shown on the sixth track.

The genome of *S. pseudocapsicum’*s chloroplast is organized in a circular form, encompassing 1,553,650 base pairs as depicted in Figure 2 and Figure S1. It is composed of four distinct sections: a major single-copy region (LSC) that spans 862,600 base pairs, a minor single-copy region (SSC) that extends for 183,250 base pairs, and a pair of inverted repeat (IR) regions, each measuring 253,900 base pairs. The total G + C content of the chloroplast genome is 37.59%, with the IR regions exhibiting the highest concentration at 43.03%, exceeding the G + C content of both the LSC and SSC regions, which are 35.68% and 31.53%, respectively. The genome contains 127 genes, including 82 protein-coding genes, 37 tRNA genes, and 8 rRNA genes, with 18 genes duplicated in the IR regions. The chloroplast genome sequencing depth distribution is illustrated in Figure S2 and the structures of the cis- and trans-splicing genes are illustrated in Figure S3.

**Figure 3.**
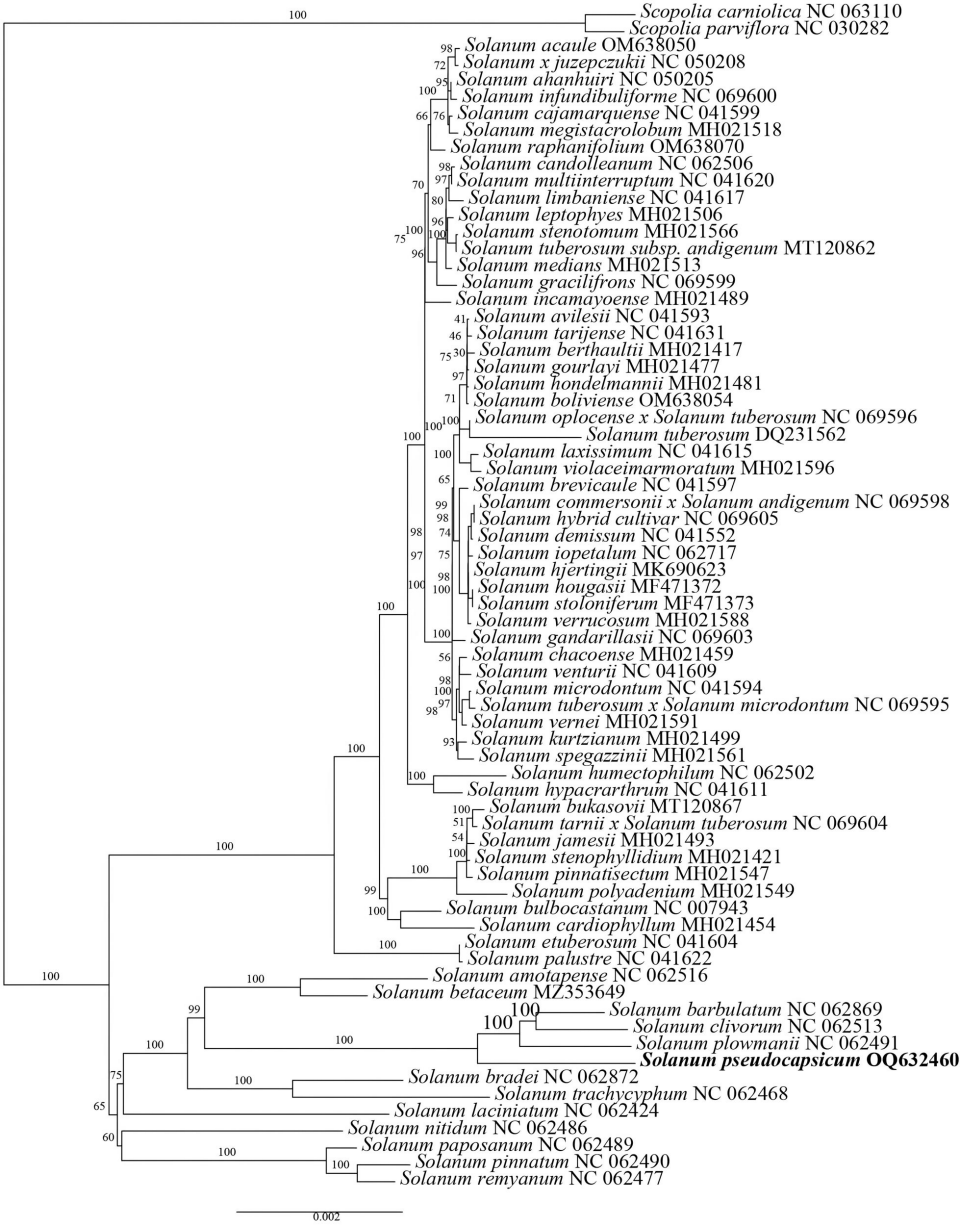
Maximum-likelihood (ML) tree of *Solanum pseudocapsicum* and 69 relative species was reconstructed using the IQ-Tree based on 68 protein-coding genes shared by all genomes. Bootstrap values are shown next to the nodes. The following sequences, of which some existed in NCBI database but were unpublished, were used:*Scopolia carniolica* NC063110 (Gandini et al. 2021), *Scopolia parviflora* NC030282 (Park and Lee 2016), *Solanum acaule* OM638050 (Park 2021), *Solanum ahanhuiri* NC050205, *Solanum amotapense* NC062516, *Solanum avilesii* NC041593, *Solanum barbulatum* NC062869, *Solanum berthaultii* MH021417 (Park 2017), *Solanum betaceum* MZ353649 (Bohs and Olmstead 1997), *Solanum boliviense* OM638054 (Yan et al. 2023), *Solanum bradei* NC062872, *Solanum brevicaule* NC041597 (Park 2019), *Solanum bukasovii* MT120867 (Achakkagari et al. 2020), *Solanum bulbocastanum* NC007943 (Daniell et al. 2006), *Solanum cajamarquense* NC041599, *Solanum candolleanum* NC062506 (Hardigan et al. 2017), *Solanum cardiophyllum* MH021454 (Rodriguez and Spooner 1997), *Solanum chacoense* MH021459 (Cho et al. 2017), *Solanum clivorum* NC062513, *Solanum commersonii x Solanum andigenum* NC069598, *Solanum demissum* NC041552 (Cho et al. 2019), *Solanum etuberosum* NC041604, *Solanum gandarillasii* NC069603, *Solanum gourlayi* MH021477, *Solanum gracilifrons* NC069599, *Solanum hjertingii* MK690623 (Park 2022), *Solanum hondelmannii* MH021481, *Solanum hougasii* MF471372 (Cho et al. 2018), *Solanum humectophilum* NC062502, *Solanum hybrid cultivar* NC069605, *Solanum hypacrarthrum* NC041611, *Solanum incamayoense* MH021489, *Solanum infundibuliforme* NC069600, *Solanum iopetalum* NC062717 (Park 2023), *Solanum jamesii* MH021493, *Solanum kurtzianum* MH021499, *Solanum laciniatum* NC062424, *Solanum laxissimum* NC041615, *Solanum leptophyes* MH021506, *Solanum limbaniense* NC041617, *Solanum medians* MH021513, *Solanum megistacrolobum* MH021518 (Ugent 1970), *Solanum microdontum* NC041594, *Solanum multiinterruptum* NC041620, *Solanum nitidum* NC062486 (Knapp 1989), *Solanum oplocense x Solanum tuberosum* NC069596, *Solanum palustre* NC041622, *Solanum paposanum* NC062489, *Solanum pinnatisectum* MH021547 (Cho and Park 2016), *Solanum pinnatum* NC062490, *Solanum plowmanii* NC062491, *Solanum polyadenium* MH021549, *Solanum raphanifolium* OM638070 (Spooner et al. 1991), *Solanum remyanum* NC062477, *Solanum spegazzinii* MH021561, *Solanum stenophyllidium* MH021421, *Solanum stenotomum* MH021566, *Solanum stoloniferum* MF471373 (Park 2018), *Solanum tarijense* NC041631, *Solanum trachycyphu*m NC062468, *Solanum tuberosum* DQ231562 (Chung et al. 2006), *Solanum venturii* NC041609, *Solanum vernei* MH021591, *Solanum verrucosum* MH021588, *Solanum violaceimarmoratum* MH021596, *Solanum x juzepczukii* NC050208, *Solanum tarnii x Solanum tuberosum* NC069604, *Solanum tuberosum subsp. Mandigenum* MT120862, *Solanum tuberosum x Solanum microdontum* NC069595.

A comprehensive phylogenetic tree, based on the chloroplast genome of *S. pseudocapsicum*, is presented in Figure 3, revealing its phylogenetic position. The analysis reveals that *S. brevicaule*, *S. pinnatisectum*, *S. etuberosum*, and *S. bulbocastanum* are genetically close and form a strongly supported clade, consistent with previous findings on the phylogenetic relationships within the *Solanum* genus. This result supports the reliability of the phylogenetic analysis conducted in this study (Olmstead et al. 2008). Furthermore, the current study identifies *S. pseudocapsicum*, *S. betaceum*, *S. laciniatum*, and *S. nitidum* as being genetically linked and situated on a single branch, a finding that contrasts with previous research where *S. pseudocapsicum* and *S. betaceum* were not grouped together (Weese and Bohs 2007). Despite discrepancies with previous research where *S. pseudocapsicum* and *S. betaceum* were not grouped together, the present study’s finding that groups *S. pseudocapsicum*, *S. betaceum*, *S. laciniatum*, and *S. nitidum* on a single branch is supported by a higher level of bootstrap values, thus this finding offers a novel perspective into the evolutionary landscape. Although the phylogenetic relationships between *S. pseudocapsicum* and its close relatives are well-supported, some of the relationships within the clade containing *S. avilezii*, *S. tarijensel*, *S. betillonum*, *S. scarlavayi*, *S. yondoense*, and *S. boliviense* are not strongly supported, with some bootstrap values below 50. This may be due to insufficient sampling in the existing databases, which is inadequate to fully support the clear taxonomic relationships within this group. However, this particular clade is relatively distantly related to *S. pseudocapsicum*, which is the focus of this study. Therefore, it does not affect the relative phylogenetic position of *S. pseudocapsicum* and its closer relatives.

## Discussion

This study provides the first complete chloroplast genome sequence of *Solanum pseudocapsicum*, a species valued for both its ornamental and medicinal properties. Our analysis of the genome revealed a typical quadripartite structure and gene content consistent with other members of the Solanaceae family. The phylogenetic analysis based on 68 shared protein-coding genes yielded valuable insights into the evolutionary relationships within the *Solanum* genus, corroborating some previously established relationships while also uncovering novel findings.

Our results strongly support the close phylogenetic affinity of *S. pseudocapsicum* with *S. betaceum*, *S. laciniatum*, and *S. nitidum*. These species clustered together with robust bootstrap support, suggesting a shared evolutionary history. This finding deviates from some earlier studies that did not place *S. pseudocapsicum* and *S. betaceum* in close proximity (Weese and Bohs 2007). However, the high bootstrap values obtained in our analysis lend strong confidence to this new grouping, potentially refining our understanding of the evolutionary relationships within this section of the *Solanum* genus.

Interestingly, our phylogenetic reconstruction also highlighted a clade comprising *S. avilezii*, *S. tarijensel*, *S. betillonum*, *S. scarlavayi*, *S. yondoense*, and *S. boliviense* with several nodes exhibiting low bootstrap support (<50%). This may be due to insufficient sampling in the existing databases, which is inadequate to fully support the clear taxonomic relationships within this group. Expanding the dataset with more closely related species is crucial to resolve the phylogenetic relationships within this group with greater confidence. However, as this clade is relatively distant from *S. pseudocapsicum*, the uncertainty surrounding its internal structure does not impact the robust placement of our focal species and its closest relatives.

## Conclusion

This study has provided a critical foundation for future investigations into the evolution, conservation, and potential utilization of *S. pseudocapsicum*. The generated chloroplast genome sequence serves as a valuable resource for understanding the phylogenetic relationships within the *Solanum* genus and contributes to the growing body of genomic data for this economically and ecologically important plant family. Further research incorporating broader sampling and multi-locus approaches will undoubtedly refine the understanding of the evolutionary history of *S. pseudocapsicum* and its relatives, ultimately contributing to a more comprehensive picture of *Solanum* diversification.

## Supplementary Material

Supplemental Material.docx

## Data Availability

The complete chloroplast genome sequence of *S. pseudocapsicum* has been deposited in the GenBank database under the accession number OQ632460 or NC077636 (these numbers were automatically generated by NCBI and refer to the same sample). The associated BioProject, SRA, and Bio-Sample numberss are PRJNA1097352, SRR28591396, and SAMN40869204, respectively.

## References

[CIT0001] Achakkagari SR, Kyriakidou M, Tai HH, Anglin NL, Ellis D, Strömvik MV. 2020. Complete plastome assemblies from a panel of 13 diverse potato taxa. PLoS One. 15(10):e0240124. doi:10.1371/journal.pone.0240124. 33031462 PMC7544113

[CIT0002] Aliero AA, Grierson DS, Afolayan AJ. 2006. The foliar micromorphology of *Solanum pseudocapsicum*. Flora-Morphol Distribut Funct Ecol Plants. 201(4):326–330. doi:10.1016/j.flora.2005.10.001.

[CIT0003] Bohs L, Olmstead RG. 1997. Phylogenetic relationships in *Solanum* (Solanaceae) based on ndhF sequences. Systematic Botany. 22(1):5–17. doi:10.2307/2419674.

[CIT0004] Chen S, Zhou Y, Chen Y, Gu J. 2018. fastp: an ultra-fast all-in-one FASTQ preprocessor. Bioinformatics. 34(17):i884–i890. doi:10.1093/bioinformatics/bty560. 30423086 PMC6129281

[CIT0005] Cho K-S, Cho J-H, Im J-S, Choi J-G, Park Y-E, Jang D-C, Hong S-Y, Park T-H. 2018. The complete chloroplast genome sequence of *Solanum hougasii*, one of the potato wild relative species. Mitochondrial DNA B Resour. 3(2):755–757. doi:10.1080/23802359.2018.1491342. 33474312 PMC7800044

[CIT0006] Cho KS, Cho JH, Park YE, Park TH. 2019. Chloroplast genome sequence of *Solanum demissum*, a wild tuber-bearing species was completed. Mitochondrial DNA Part B. 4(1):1800–1802. doi:10.1080/23802359.2019.1612716.

[CIT0007] Cho KS, Choi JG, Cho JH, Im JS, Park YE, Hong SY, Park TH. 2017. Chloroplast genome of the wild tuber-bearing diploid potato relative *Solanum chacoense*. Mitochondrial DNA B Resour. 2(2):915–917. doi:10.1080/23802359.2017.1413309. 33474034 PMC7799535

[CIT0008] Cho KS, Park TH. 2016. Complete chloroplast genome sequence of *Solanum nigrum* and development of markers for the discrimination of *S. nigrum*. Hortic Environ Biotechnol. 57(1):69–78. doi:10.1007/s13580-016-0003-2.

[CIT0009] Chung H-J, Jung JD, Park H-W, Kim J-H, Cha HW, Min SR, Jeong W-J, Liu JR. 2006. The complete chloroplast genome sequences of *Solanum tuberosum* and comparative analysis with *Solanaceae species* identified the presence of a 241-bp deletion in cultivated potato chloroplast DNA sequence. Plant Cell Rep. 25(12):1369–1379. doi:10.1007/s00299-006-0196-4. 16835751

[CIT0010] Daniell H, Lee S-B, Grevich J, Saski C, Quesada-Vargas T, Guda C, Tomkins J, Jansen RK. 2006. Complete chloroplast genome sequences of *Solanum bulbocastanum*, *Solanum lycopersicum* and comparative analyses with other Solanaceae genomes. Theor Appl Genet. 112(8):1503–1518. doi:10.1007/s00122-006-0254-x. 16575560

[CIT0011] Gandini CL, Ibañez VN, Zubko MK, Sanchez-Puerta MV. 2021. Complete plastome phylogeny and an update on cox1 intron evolution of Hyoscyameae (Solanaceae). Org Divers Evol. 21(3):521–532. doi:10.1007/s13127-021-00501-3.

[CIT0012] Guo S, Liao X, Chen S, Liao B, Guo Y, Cheng R, Xiao S, Hu H, Chen J, Pei J, et al. 2022. A comparative analysis of the chloroplast genomes of four polygonum medicinal plants. Front Genet. 13:764534. doi:10.3389/fgene.2022.764534. 35547259 PMC9084321

[CIT0013] Hardigan MA, Laimbeer FPE, Newton L, Crisovan E, Hamilton JP, Vaillancourt B, Wiegert-Rininger K, Wood JC, Douches DS, Farré EM. 2017. Genome diversity of tuber-bearing *Solanum* uncovers complex evolutionary history and targets of domestication in the cultivated potato. Proc Natl Acad Sci U S A. 114(46):E9999–E10008. doi:10.1073/pnas.1714380114. 29087343 PMC5699086

[CIT0014] Jeyasankar A, Premalatha S, Elumalai K. 2012. Biological activities of *Solanum pseudocapsicum* (Solanaceae) against cotton bollworm, Helicoverpa armigera Hübner and armyworm, Spodoptera litura Fabricius (Lepidotera: Noctuidae). Asian Pac J Trop Biomed. 2(12):981–986. doi:10.1016/S2221-1691(13)60010-6. 23593579 PMC3621475

[CIT0015] Jin JJ, Yu WB, Yang JB, Song Y, DePamphilis CW, Yi TS, Li DZ. 2020. GetOrganelle: a fast and versatile toolkit for accurate de novo assembly of organelle genomes. Genome Biol. 21(1):241. doi:10.1186/s13059-020-02154-5. 32912315 PMC7488116

[CIT0016] Knapp S. 1989. A revision of the *Solanum nitidum* group (section Holophylla pro parte): Solanaceae. Bulletin of the British Museum. Natural History. Botany. 19:63–102.

[CIT0017] Nguyen LT, Schmidt HA, Von Haeseler A, Minh BQ. 2015. IQ-TREE: a fast and effective stochastic algorithm for estimating maximum-likelihood phylogenies. Mol Biol Evol. 32(1):268–274. doi:10.1093/molbev/msu300. 25371430 PMC4271533

[CIT0018] Olmstead RG, Bohs L, Migid HA, Santiago-Valentin E, Garcia VF, Collier SM. 2008. A molecular phylogeny of the Solanaceae. Taxon. 57(4):1159–1181. doi:10.1002/tax.574010.

[CIT0019] Park JH, Lee J. 2016. The complete plastid genome of *Scopolia parviflora (Dunn.) Nakai* (Solanaceae). Korean J Plant Taxonomy. 46(1):60–64. doi:10.11110/kjpt.2016.46.1.60.

[CIT0020] Park TH. 2017. The complete chloroplast genome of *Solanum berthaultii*, one of the potato wild relative species. Mitochondrial DNA B Resour. 2(1):88–89. doi:10.1080/23802359.2017.1285213. 33473725 PMC7800855

[CIT0021] Park TH. 2018. Chloroplast genome sequence of the wild tetraploid potato relative *Solanum stoloniferum*. Mitochondrial DNA B Resour. 3(1):416–418. doi:10.1080/23802359.2018.1456983. 33474189 PMC7799944

[CIT0022] Park TH. 2019. Complete chloroplast genome sequence of the wild diploid potato relative, *Solanum brevicaule*. Mitochondrial DNA B Resour. 4(2):4159–4160. doi:10.1080/23802359.2019.1693292. 33366362 PMC7707683

[CIT0023] Park TH. 2021. Complete chloroplast genome sequence of the wild diploid potato relative, *Solanum acaule*. Mitochondrial DNA B Resour. 6(3):1189–1191. doi:10.1080/23802359.2021.1902414. 33829083 PMC8008929

[CIT0024] Park TH. 2022. Complete chloroplast genome sequence of *Solanum hjertingii*, one of the wild potato relatives. Mitochondrial DNA B Resour. 7(4):715–717. doi:10.1080/23802359.2022.2068983. 35493716 PMC9045759

[CIT0025] Park TH. 2023. Complete chloroplast genome sequence of *Solanum iopetalum*, one of the tuber-bearing wild potato relatives. Mitochondrial DNA B Resour. 8(3):347–351. doi:10.1080/23802359.2023.2183720. 36876142 PMC9980020

[CIT0026] Rodriguez A, Spooner DM. 1997. Chloroplast DNA analysis of *Solanum bulbocastanum* and *S. cardiophyllum*, and evidence for the distinctiveness of *S. cardiophyllum subsp. ehrenbergii (sect. Petota).* Systematic Botany. 22(1):31–43. doi:10.2307/2419676.

[CIT0027] Rozewicki J, Li S, Amada KM, Standley DM, Katoh K. 2019. MAFFT-DASH: integrated protein sequence and structural alignment. Nucleic Acids Res. 47(W1):W5–W10. doi:10.1093/nar/gkz342. 31062021 PMC6602451

[CIT0028] Shi L, Chen H, Jiang M, Wang L, Wu X, Huang L, Liu C. 2019. CPGAVAS2, an integrated plastome sequence annotator and analyzer. Nucleic Acids Res. 47(W1):W65–W73. doi:10.1093/nar/gkz345. 31066451 PMC6602467

[CIT0029] Spooner DM, Sytsma KJ, Smith JF. 1991. A molecular reexamination of diploid hybrid speciation of *Solanum raphanifolium*. Evolution. 45(3):757–764. doi:10.1111/j.1558-5646.1991.tb04344.x. 28568823

[CIT0030] Ugent D. 1970. The Potato: What is the botanical origin of this important crop plant, and how did it first become domesticated? Science. 170(3963):1161–1166. doi:10.1126/science.170.3963.1161. 17744042

[CIT0031] Vijayan P, Vijayaraj P, Setty PHC, Hariharpura RC, Godavarthi A, Badami S, Arumugam DS, Bhojraj S. 2004. The cytotoxic activity of the total alkaloids isolated from different parts of *Solanum pseudocapsicum*. Biol Pharm Bull. 27(4):528–530. doi:10.1248/bpb.27.528. 15056860

[CIT0032] Weese TL, Bohs L. 2007. A three-gene phylogeny of the genus *Solanum* (Solanaceae). Issn: 0363-6445. 32(2):445–463. doi:10.1600/036364407781179671.

[CIT0033] Wu ZY, Raven PH, Hong DY. 1994. Flora of China. Vol 17 (Lamiaceae through Verbenaceae,). St. Louis: Science Press, Beijing, and Missouri Botanical Garden Press.

[CIT0034] Xu H, Xu L, Yang P, Cao Y, Tang Y, He G, Yuan S, Ming J. 2018. Tobacco rattle virus-induced PHYTOENE DESATURASE (PDS) and Mg-chelatase H subunit (ChlH) gene silencing in *Solanum pseudocapsicum L.* PeerJ. 6:e4424. doi:10.7717/peerj.4424. 29576941 PMC5865466

[CIT0035] Yan L-J, Zhu Z-G, Wang P, Fu C-N, Guan X-J, Kear P, Zhang C-Z, Zhu G-T. 2023. Comparative analysis of 343 plastid genomes of *Solanum* section Petota: insights into potato diversity, phylogeny, and species discrimination. J of Sytematics Evolution. 61(4):599–612. doi:10.1111/jse.12898.

[CIT0036] Zhang N, Xie P, Huang K, Yin H, Mo P, Wang Y. 2023. The complete chloroplast genome sequence of Centaurea cyanus (Asteraceae). Mitochondrial DNA B Resour. 8(3):393–397. doi:10.1080/23802359.2023.2185470. 36926644 PMC10013558

