# Supplementary material for: Characterization and phylogenetic analysis of the chloroplast genome of Solanum pseudocapsicum (Solanaceae)
Source: Mitochondrial DNA B Resour. 2024 Sep 30;9(10):1285–90. doi: 10.1080/23802359.2024.2410442 (PMC11443543; doi:10.1080/23802359.2024.2410442)
Supplement: Supplemental Material.docx [file TMDN_A_2410442_SM2653.docx]

**Supplemental Material**

**
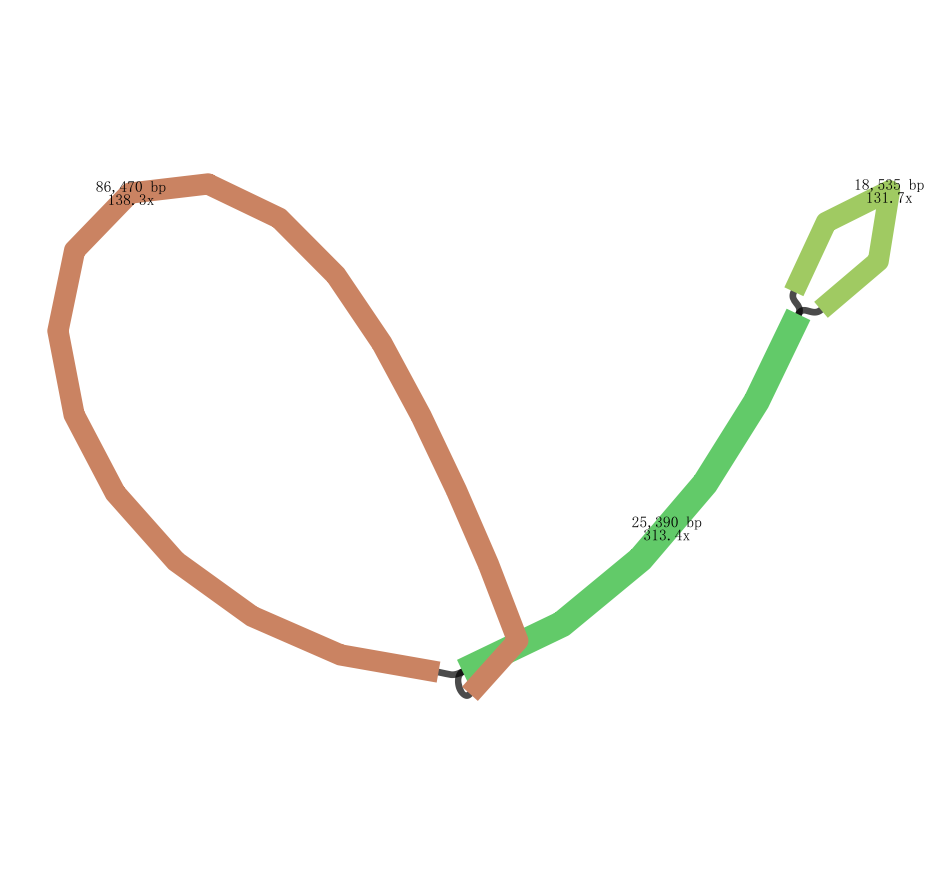
**

**Figure S1.** The schematic representation of the coverage depth for the entire chloroplast genome of *Solanum pseudocapsicum* using Bandage. The numbers indicate the depths of different regions.


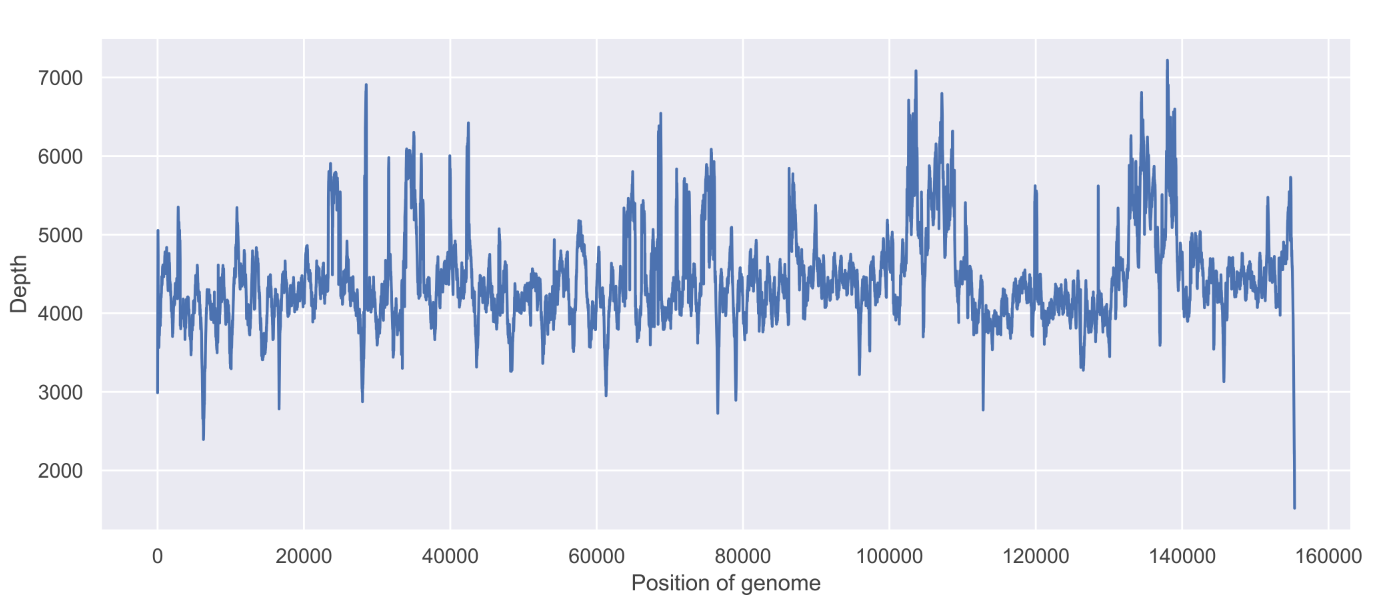


Figure S2. Chloroplast genome sequencing depth distribution for *Solanum pseudocapsicum*. The graph illustrates the chloroplast genome sequencing depth distribution for species *Elaeocarpus duclouxii*, with the horizontal axis representing genomic position and the vertical axis indicating sequencing depth.

**
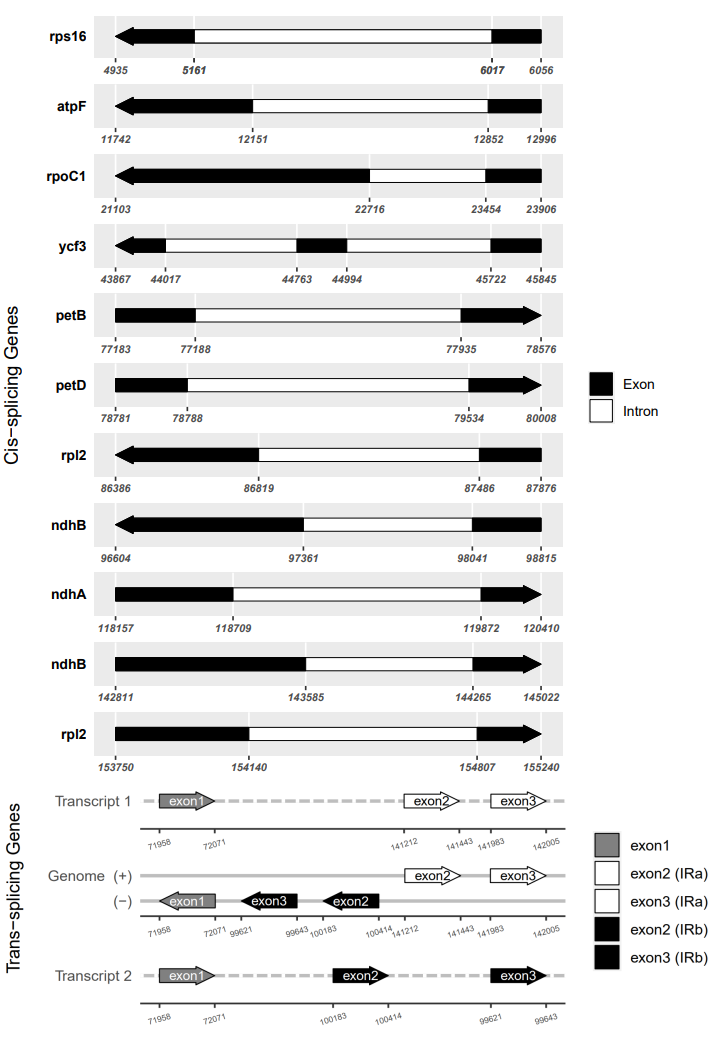
**

**Figure S3.** Schematic map of the cis-splicing genes and trans-splicing gene rps12 in the chloroplast genome of *Solanum pseudocapsicum* using CPGView. The exons of the cis-splicing genes are shown in black; the introns are shown in white. The arrow indicates the sense direction of the gene. Please note that lengths of exons and introns are not drawn to scale.
